# Cytochrome‐mediated direct electron uptake from metallic iron by *Methanosarcina acetivorans*

**DOI:** 10.1002/mlf2.12044

**Published:** 2022-11-17

**Authors:** Dawn E. Holmes, Haiyan Tang, Trevor Woodard, Dandan Liang, Jinjie Zhou, Xinying Liu, Derek R. Lovley

**Affiliations:** ^1^ Department of Microbiology University of Massachusetts‐Amherst Amherst Massachusetts USA; ^2^ Department of Physical and Biological Science Western New England University Springfield Massachusetts USA; ^3^ Jiangsu Provincial Key Lab of Solid Organic Waste Utilization, Jiangsu Collaborative Innovation Center of Solid Organic Wastes, Educational Ministry Engineering Center of Resource‐saving Fertilizers Nanjing Agricultural University Nanjing China; ^4^ Present address: State Key Laboratory of Urban Water Resource and Environment, School of Environment Harbin Institute of Technology Harbin China; ^5^ Present address: Institute for Advanced Study Shenzhen University Shenzhen Guangdong China; ^6^ Present address: College of Environmental Science and Engineering Beijing Forestry University Beijing China

## Abstract

Methane‐producing microorganisms accelerate the corrosion of iron‐containing metals. Previous studies have inferred that some methanogens might directly accept electrons from Fe(0), but when this possibility was more intensively investigated, H_2_ was shown to be an intermediary electron carrier between Fe(0) and methanogens. Here, we report that *Methanosarcina acetivorans* catalyzes direct metal‐to‐microbe electron transfer to support methane production. Deletion of the gene for the multiheme, outer‐surface *c*‐type cytochrome MmcA eliminated methane production from Fe(0), consistent with the key role of MmcA in other forms of extracellular electron exchange. These findings, coupled with the previous demonstration that outer‐surface *c*‐type cytochromes are also electrical contacts for electron uptake from Fe(0) by *Geobacter* and *Shewanella* species, suggest that the presence of multiheme *c*‐type cytochromes on corrosion surfaces might be diagnostic for direct metal‐to‐microbe electron transfer and that interfering with cytochrome function might be a strategy to mitigate corrosion.

1

Anaerobic microbial activity can accelerate the corrosion of iron‐containing metals, a substantial economic concern^1^. Most studies of microbial corrosion have focused on sulfate‐reducing bacteria because anoxic sulfate‐rich environments are particularly corrosive^2^, ^3^. However, methane‐producing archaea are often abundant on the surfaces of corroding iron metals and several methanogen isolates promote corrosion^4^, ^5^, ^6^, ^7^, ^8^.

In previous studies with pure cultures^6^, ^7^, methanogens primarily accelerated corrosion by consuming H_2_ produced from the oxidation of Fe(0) to Fe(II):

(1) $$\text{Fe}\left(0\right){\;+\text{2H}}^{+}\to \text{Fe}\left(\text{II}\right){\;+\;H}_{2}$$

H_2_ is abiotically produced from Fe(0)^9^ and extracellular hydrogenases can increase the rate of H_2_ production^6^, ^7^. In some instances, the hydrogenases are released from moribund cells^6^. However, some methanogens specifically express hydrogenases that are exported outside the cell^7^. Methanogens with these hydrogenases corrode Fe(0) faster than methanogens lacking the appropriate extracellular hydrogenase genes^7^.

It has also been proposed that some methanogens may extract electrons from Fe(0) via direct metal‐to‐microbe electron transfer^5^, ^8^. However, direct electron transfer was not rigorously evaluated. The possibility for direct electron uptake was inferred from the observation that these strains produced methane faster than other strains with Fe(0) as the electron donor^5^, ^8^. However, all of these strains also had the ability to use H_2_ as an electron donor, raising the possibility that other physiological properties accounted for the enhanced corrosion^10^. In fact, some methanogen strains proposed to directly extract electrons from Fe(0) were subsequently found to express extracellular hydrogenases that were responsible for their enhanced corrosion abilities^6^, ^7^.

In a similar manner, the sulfate‐reducers *Desulfovibrio ferrophilus* and *Desulfopila corrodens* were initially proposed to be capable of direct electron uptake from Fe(0) due to their rapid corrosion capability^5^. However, subsequent studies comparing sulfate reduction with pure Fe(0), which abiotically produces H_2_, and stainless steel, which does not, indicated that these sulfate reducers rely on H_2_ as an intermediary electron carrier when accepting electrons from Fe(0)^11^.

*Geobacter* and *Shewanella* species are the only microbes for which there is strong evidence for direct metal‐to‐microbe electron transfer. Strains of *Geobacter sulfurreducens* and *Geobacter metallireducens* unable to use H_2_ as an electron donor were able to use pure Fe(0) as the sole electron donor supporting anaerobic respiration^9^, ^11^, ^12^. Deletion of genes for outer‐surface, multiheme *c*‐type cytochromes known to be important for extracellular electron exchange with other acceptors/donors inhibited electron uptake from the iron metals^9^, ^12^. Evidence for the ability of *Shewanella oneidensis* to consume electrons via direct microbe‐to‐metal electron transfer was the finding that deleting the genes for its primary porin–cytochrome conduit inhibited corrosion of stainless steel by biofilms that had been grown on organic‐rich media with oxygen as the electron acceptor^13^. However, oxygen, or the fumarate, nitrate, or Fe(III) that *Geobacter* or *Shewanella* species employ as electron acceptors for Fe(0) oxidation might not be abundant near the surface of most corroding metal surfaces. In contrast, carbon dioxide, the electron acceptor for methanogenesis, is ubiquitous.

*Methanosarcina acetivorans* is a genetically tractable representative of Type II *Methanosarcina* ^14^, which does not consume H_2_ ^15^. Genetic and biochemical studies suggest that its seven‐heme membrane‐associated *c*‐type cytochrome MmcA is an outer surface electrical contact for electron transfer to extracellular electron acceptors such as the humics analog anthraquinone 2,6‐disulfonate (AQDS) and Fe(III)^16^, ^17^ as well as direct electron uptake from other microbes^18^. Less than 90 μM methane was produced when *M. acetivorans* was incubated with pure Fe(0) as the sole potential electron donor (Figure 1A). However, when the Fe(0)‐containing medium was supplemented with 1 mM acetate, methane was produced well in excess of the ca. 1 mM methane generated in the presence of acetate alone (Figure 1A). Methane production in the presence of Fe(0) and acetate stopped at about the same time that methane production stopped in acetate‐only controls. Notably, this was the point at which the additional 1 mM acetate was stoichiometrically converted to about 1 mM methane (Figure 1A). These results suggest that *M. acetivorans* requires concurrent acetate conversion to methane as an additional energy source to extract electrons from Fe(0) for methane production.

**Figure 1:**
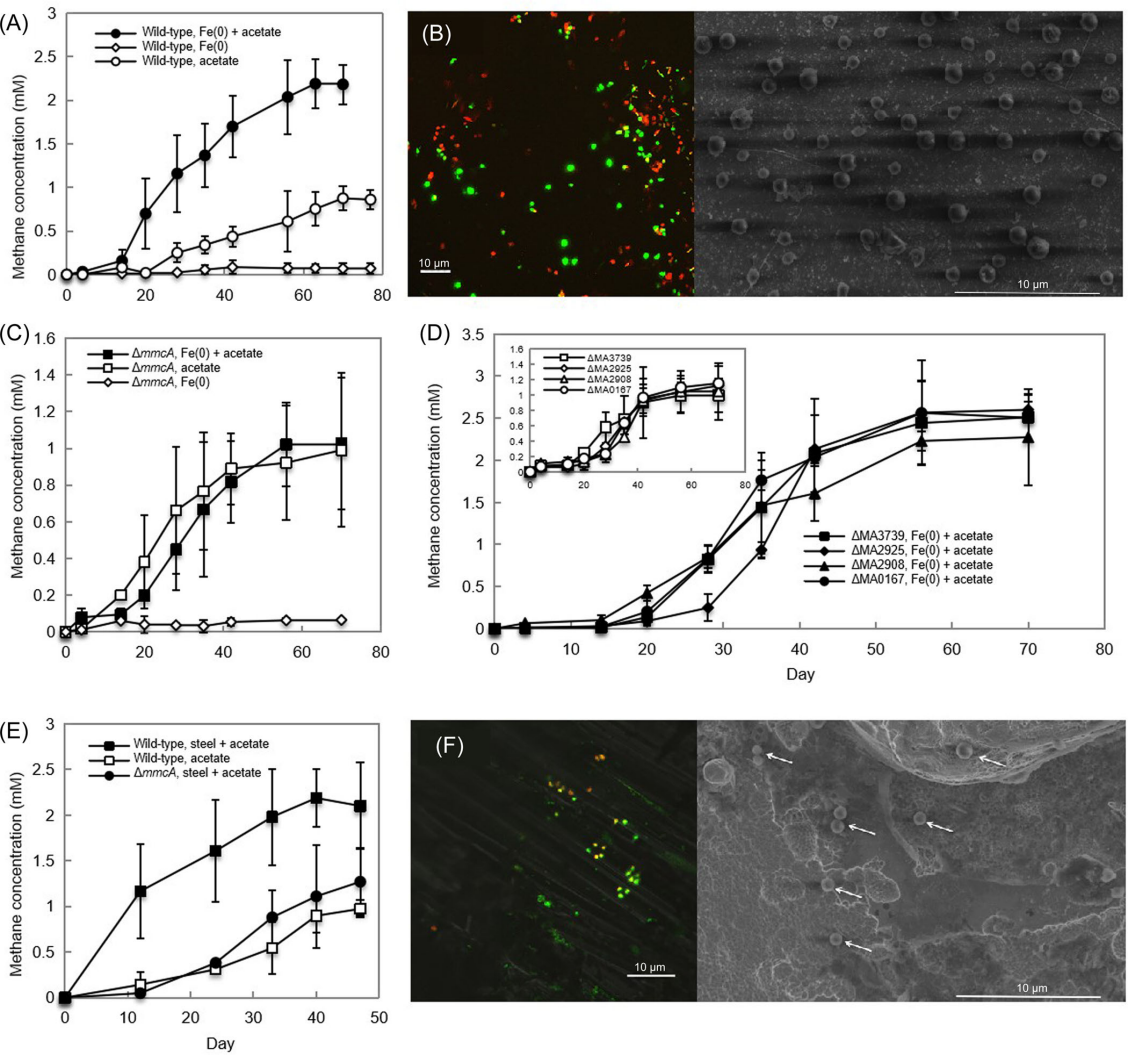
Methane production from pure Fe(0) and stainless steel by wild‐type and cytochrome‐deficient strains of *Methanosarcina acetivorans*. (A) Methane production by wild‐type *M. acetivorans* grown with Fe(0) granules (1–2 mm, 2 g per 50 ml of culture) as the electron donor in the presence or absence of 1 mM acetate. (B) Confocal laser scanning microscopy (CLSM) and scanning electron microscope (SEM) images of *M. acetivorans* growing on the surface of a Fe(0) particle. Cells were treated with Live/Dead stain for CLSM. (C) Methane production by the Δ*mmcA* cytochrome deletion strain of *M. acetivorans* in the presence of 1 mM acetate with or without added Fe(0). (D) Methane production by ΔMA3739, ΔMA2925, ΔMA2908, and ΔMA0167 cytochrome deletion strains of *M. acetivorans* in the presence of Fe(0) and 1 mM acetate. Inset: Methane production with only 1 mM acetate. (E) Methane production by wild‐type and Δ*mmcA* strains of *M. acetivorans* grown in the presence of stainless steel cubes and 1 mM acetate as well as wild‐type with acetate alone. (F) CLSM and SEM images of *M. acetivorans* cells growing on the surface of a stainless steel cube. The white arrows in the SEM image point to cells. Methodological details are provided in the Supporting Information. All error bars represent the standard deviation from triplicate cultures.

Oxidation of Fe(0) generates Fe^2+^, which precipitates as carbonate minerals in the bicarbonate‐buffered media, but some Fe^2+^ remains in the solution. In accordance with *M. acetivorans* consuming electrons from Fe(0), soluble Fe^2+^ accumulated over time in *M. acetivorans* cultures producing methane in the presence of Fe(0) and acetate, but not in abiotic controls or controls amended with culture filtrates, which did not produce methane (Figure S1).

The inability of *M*. *acetivorans* to use H_2_ as an electron donor ruled out the possibility that H_2_ served as an intermediary electron carrier from Fe(0) for methane production. Fe(0) did not generate carbon monoxide as a potential electron carrier. The N_2_/CO_2_ gas mixture overlying the medium contained 1.5 × 10^−7^ atm of carbon monoxide and there was no increase in carbon monoxide concentration over time in sterile controls, cultures of *M. acetivorans* growing on Fe(0), or when filtrates of cultures grown on Fe(0) were added to Fe(0) (Figure S2).

A lack of culture turbidity indicated that planktonic cell growth was not significant. Both confocal and scanning electron microscopy revealed that attached cells were scattered over the Fe(0) surface (Figure 1B). All of the cells were in direct contact with the surface, and no cell‐on‐cell stacking within biofilms was observed. Thus, cells were appropriately positioned for direct metal‐to‐microbe electron transfer.

*M. acetivorans* requires the outer‐surface *c*‐type cytochrome MmcA for direct electron uptake from *G. metallireducens* ^18^. A MmcA‐deficient mutant of *M. acetivorans* was defective in methane production from Fe(0). In the presence of Fe(0) and 1 mM acetate, the MmcA‐deficient mutant only produced nearly 1 mM methane, the same amount of methane produced in the wild‐type acetate‐only control (Figure 1A and C). MmcA is not required for methane production from acetate^16^. Thus, the reduced methane production from the combination of Fe(0) and acetate can be attributed to the inability of the MmcA‐deficient mutant to extract electrons from Fe(0).

Deletion of the four other *c*‐type cytochrome genes present in the *M*. *acetivorans* genome (MA3739, MA0167, MA2925, and MA2908) had no impact on the production of methane from Fe(0) (Figure 1D). This is consistent with previous results that demonstrated that none of these cytochromes was required for direct electron uptake from *G. metallireducens*.^18^ In each case, the mutant strains produced substantially more methane in the presence of Fe(0) and acetate than in controls with acetate alone (Figure 1D), in a manner comparable to wild‐type cells (Figure 1A). These results further suggest that as with direct electron uptake from *G. metallireducens*, *M. acetivorans* specifically requires MmcA as an electron carrier for electron uptake from Fe(0).

Stainless steel is much more resistant to corrosion than pure Fe(0), in part because it does not abiotically react with protons to produce H_2_ at a circumneutral pH^12^. As noted above for pure Fe(0), there was also no carbon monoxide generated from stainless steel. However, *M. acetivorans* was able to extract electrons from stainless steel and produce methane in excess of the methane attributed to acetate metabolism, which was comparable to the excess methane production observed with pure Fe(0) (Figure 1E). The MmcA‐deficient mutant only produced ~1 mM methane that was generated in the acetate‐only controls (Figure 1E). These results further demonstrate the importance of MmcA in electron uptake from Fe(0). Cells grew in contact with the stainless steel, scattered across the surface in a manner similar to that observed with pure Fe(0) (Figure 1F).

*M. acetivorans* is the first methanogen for which there is strong evidence for direct metal‐to‐microbe electron transfer. The most common route for pure culture isolates to promote corrosion is electron transfer from Fe(0) with H_2_ serving as an intermediary electron shuttle^10^. An H_2_ intermediate could be ruled out for *M. acetivorans* corrosion because: (1) *M. acetivorans* cannot use H_2_ as an electron donor^15^; (2) *M. acetivorans* derived electrons to support methane production from stainless steel, a source of Fe(0) that does not generate H_2_; and (3) methane was not produced by a strain lacking MmcA, a known electrical contact for extracellular electron exchange, a phenotype inconsistent with an H_2_ intermediate.

*M. acetivorans* only produced methane with electrons derived from Fe(0) when acetate was available as an additional energy source. The reasons for this requirement are not readily apparent from existing knowledge of *M. acetivorans* physiology and will require further investigation. One possibility is that *M. acetivorans* is unable to generate the required biosynthetic components with carbon dioxide as the only carbon source. However, providing casamino acids as a source of fixed carbon did not enable *M. acetivorans* to grow on Fe(0) in the absence of acetate. The *M. acetivorans* energy yield from direct electron uptake coupled to carbon dioxide reduction may be small^18^. During direct interspecies electron transfer (DIET), the energy available from processing electrons received from the electron‐donating partner is supplemented with the concurrent production of acetate as the organic substrate is metabolized. The lack of this additional energy source during Fe(0) oxidation may explain why acetate additions were required.

*Methanosarcinales* are often significant members of biofilms on corroded steel in anoxic low‐sulfide environments^19^, ^20^, frequently in association with acetogens^20^. In the absence of substantial organic matter metabolism to generate acetate, Type II *Methanosarcina*, like *M. acetivorans*, may require the acetate generated by acetogens from Fe(0) to directly accept electrons from Fe(0).

The finding that outer‐surface multiheme cytochromes are key electrical contacts for direct metal‐to‐microbe electron transfer in *M. acetivorans* as well as *Geobacter* ^9^, ^12^ and *Shewanella* ^13^ species suggests that metagenomic mining for similar cytochrome sequences may aid in determining the relative importance of direct electron uptake on corroding surfaces versus other corrosion mechanisms, such as enhanced H_2_ production with extracellular hydrogenases^4^ that may also be revealed with molecular analyses. Expanding corrosion studies to a wider diversity of microbes seems likely to not only advance the molecular diagnosis of corrosion mechanisms but also suggest novel strategies for corrosion mitigation.

## AUTHOR CONTRIBUTIONS

Dawn E. Holmes conducted experiments, organized the study, and wrote the manuscript. Haiyan Tang took the SEM images. Trevor Woodard took the confocal images. Dandan Liang, Jinjie Zhou, and Xinying Liu helped with the culturing. Derek R. Lovley helped to organize the study and to write the manuscript.

## ETHICS STATEMENT

No animal or human research was involved in this study.

## CONFLICT OF INTERESTS

The authors declare no conflict of interests.

## Supporting information

Supporting information.

## Data Availability

The authors confirm that the data supporting the findings of this study are available within the article and its Supporting Information Materials.
