# Supplementary material for: Cytochrome‐mediated direct electron uptake from metallic iron by Methanosarcina acetivorans
Source: mLife. 2022 Nov 17;1(4):443–7. doi: 10.1002/mlf2.12044 (PMC10989991; doi:10.1002/mlf2.12044)
Supplement: Supplementary file 1 — Supporting information. [file MLF2-1-443-s001.docx]

**Supplemental File**

**Cytochrome-Mediated Direct electron uptake from Metallic Iron by *Methanosarcina acetivorans***

Dawn E Holmes^1,2^*, Haiyan Tang^1,3^, Trevor Woodard^1^, Dandan Liang^1,4^, Jinjie Zhou^1,5^, Xinying Liu^1,6^, and Derek R Lovley^1^

1. Department of Microbiology, University of Massachusetts-Amherst, Amherst, MA, USA

2. Department of Physical and Biological Science, Western New England University, Springfield, MA, USA

3. Jiangsu Provincial Key Lab of Solid Organic Waste Utilization, Jiangsu Collaborative Innovation Center of Solid Organic Wastes, Educational Ministry Engineering Center of Resource-saving Fertilizers, Nanjing Agricultural University, Nanjing, 210095, Jiangsu, China

4. Current address: State Key Laboratory of Urban Water Resource and Environment, School of Environment, Harbin Institute of Technology, Harbin 150090, P.R. China

5. Current address: Institute for Advanced Study, Shenzhen University, Shenzhen, Guangdong, China

6. Current address: College of Environmental Science and Engineering, Beijing Forestry University, Beijing, 100083, China

^*^Corresponding author

**Supplemental Results**

**(B)**

**Supplementary Figure S1**. Production of methane and dissolved Fe^+2^ by actively growing cells, filtrate, and abiotic controls. (A) Methane production and (B) soluble Fe^+2^ accumulation from cultures grown in the presence of Fe(0) or stainless steel and 1 mM acetate.

**Supplementary Figure S2.** Carbon monoxide concentrations in the headspace of: the background 80:20 N_2_:CO_2_ gas mixture used for growth of *M. acetivorans* (background); live cultures grown in the presence of Fe(0) (cells Fe(0)); filtrate from actively growing cultures incubated in the presence of Fe(0) for 24 h (filtrate Fe(0)).

**Materials and Methods**

**Culture media and growth conditions**

*Methanosarcina acetivorans* strain WWM1 (∆*hpt*) (1), and all *c*-type cytochrome deletion mutant strains (∆MA2908, ∆MA0167, ∆MA2925, ∆*mmcA* (∆MA0658), ∆MA3739) were routinely cultured under strict anaerobic conditions at 37 °C in MA medium as previously described (2). Previous transcriptomic analyses demonstrated that the mutations did not cause polar effects (3). All *Methanosarcina* cultures were incubated in an N_2_-CO_2_ atmosphere (80:20, vol/vol) with methanol (20 mM) and acetate (40 mM) provided as substrates for growth.

**Testing extracellular electron uptake capabilities**

To determine whether the methanogenic strains could utilize Fe(0) as the sole electron donor for methanogenesis, 6 stainless steel cubes or 2 g of Fe(0) granules (1-2 mm; Thermo Scientific Chemicals) were supplied as electron donors in 50 ml MA medium under an N_2_-CO_2_ atmosphere. The 316L stainless steel was obtained from the Institute of Metal Research, Chinese Academy of Sciences (Shenyang, China) and was cut into cubes (2 mm × 3 mm × 3 mm). The stainless steel cubes and Fe(0) granules were pretreated as previously described (4, 5). When noted, 1 mM acetate was added to the MA medium as an additional substrate.

To evaluate the possibility that cells might release factors that would promote the generation of carbon monoxide from pure Fe(0) or stainless steel, supernatants of cultures grown on the relevant iron source were anaerobically filtered through sterile 0.2 µm syringe filters (Corning Inc, NY). The culture filtrates were added to sterile tubes of media containing the same iron metal from which the culture supernatants were collected.

**Analytical techniques**

Methane in the headspace was measured by gas chromatography with a flame ionization detector (Shimadzu, GC-8A) as previously described (6), and acetate concentrations were measured with a SHIMADZU high performance liquid chromatograph (HPLC) with an AminexTM HPX-87H Ion Exclusion column (300 mm × 7.8 mm) and an eluent of 8.0 mM sulfuric acid. Carbon monoxide was measured on a ta3000R-N2 Gas Analyzer with a detection limit of 10^-8^ atm with N_2_ as the carrier gas. Dissolved Fe^+2^ in the culture supernatant was determined with the ferrozine assay as previously described (7).

**Scanning electron microscopy**

Fe(0) particles were collected from cultures when methane production plateaued and fixed with 2.5% glutaraldehyde in 0.1 M phosphate buffer for 12 hours at 4°C. They were then washed 3 times in 0.1 M phosphate buffer at 4°C for 10 min and dehydrated in successive ethanol/water mixtures of 35%, 50%, 70%, 80%, 90%, 95% and 100% for 10 minutes. The 100% ethanol step was repeated 3 times. Samples were further dehydrated in pure hexamethyldisilazane (Sigma Aldrich, St Louis, MO, USA) for 3 min at room temperature, and dried with a stream of high purity nitrogen for 30 minutes. Scanning electron microscopy was conducted with an ultra-high resolution field emission scanning electron microscope (FEI Magellan 400, Nanolab Technologies, CA, USA).

**Confocal microscopy**

Metals for confocal microscopy were removed, washed in isotonic wash buffer for 10 minutes, stained with the Live/Dead *Bac*Light Bacterial Viability kit (ThermoFisher) (2mL staining, 3µL each stain per mL wash buffer) for 10 minutes, destained with wash buffer for 10 minutes, and mounted on petri plates with built-in cover slips with antifade reagent in glycerol.  Cells were visualized on a Nikon A1R-SIMe confocal microscope with FITC/TRITC, and transmitted light as needed, using 60X and 100X objectives.

**References**

1. Pritchett MA, Zhang JK, Metcalf WW. 2004. Development of a markerless genetic exchange method for *Methanosarcina acetivorans* C2A and its use in construction of new genetic tools for methanogenic archaea. Appl Environ Microbiol 70:1425-33.

2. Holmes D, Zhou J, Ueki T, Woodard T, Lovley D. 2021. Mechanisms for electron uptake by *Methanosarcina acetivorans* during direct interspecies electron transfer. mBio 12:e02344-21.

3. Holmes DE, Ueki T, Tang HY, Zhou J, Smith JA, Chaput G, Lovley DR. 2019. A Membrane-Bound Cytochrome Enables *Methanosarcina acetivorans* To Conserve Energy from Extracellular Electron Transfer. MBio 10:e00789-19.

4. Tang HY, Holmes DE, Ueki T, Palacios PA, Lovley DR. 2019. Iron Corrosion via Direct Metal-Microbe Electron Transfer. mBio 10:e00303-19

5. Tang HY, Yang C, Ueki T, Pittman CC, Xu D, Woodard TL, Holmes DE, Gu T, Wang F, Lovley DR. 2021. Stainless steel corrosion via direct iron-to-microbe electron transfer by *Geobacter* species. ISME J 15:3084-3093.

6. Holmes DE, Giloteaux L, Orellana R, Williams KH, Robbins MJ, Lovley DR. 2014. Methane production from protozoan endosymbionts following stimulation of microbial metabolism within subsurface sediments. Frontiers in Microbiology 5:366.

7. Lovley DR, Phillips EJ. 1987. Rapid assay for microbially reducible ferric iron in aquatic sediments. Appl Environ Microbiol 53:1536-40.
